# Characterizations of Cancer Gene Mutations in Chinese Metastatic Breast Cancer Patients

**DOI:** 10.3389/fonc.2020.01023

**Published:** 2020-06-30

**Authors:** Zhonghua Tao, Ting Li, Zhe Feng, Chang Liu, Yilin Shao, Mingyu Zhu, Chengcheng Gong, Biyun Wang, Jun Cao, Leipin Wang, Yiqun Du, Analyn Lizaso, Bing Li, Jian Zhang, Xichun Hu

**Affiliations:** ^1^Department of Medical Oncology, Fudan University Shanghai Cancer Center, Shanghai, China; ^2^Department of Oncology, Shanghai Medical College, Fudan University, Shanghai, China; ^3^Burning Rock Biotech, Guangzhou, China

**Keywords:** genomic profiling, metastatic breast cancer, liquid biopsy, next-generation sequencing, ctDNA assay

## Abstract

**Background:** Breast cancer (BC) is a type of disease with high heterogeneity. Molecular profiling, by revealing the intrinsic nature of its various subtypes, has extensively improved the therapeutic management of BC patients. However, the genomic mutation landscape of Chinese metastatic BC has not been fully explored.

**Methods:** Matched plasma and mononuclear cells from 290 Chinese women with metastatic BC were sequenced using either of the two commercially-available panels consisting of 520 cancer-related and 108 BC-related genes. Both panels cover the same critical regions of 91 genes. The circulating tumor DNA mutation profile from our cohort was then compared with publicly-available metastatic BC datasets from Memorial Sloan Kettering Cancer Center (MSKCC) and Pan-cancer analysis of whole genomes (PCAWG).

**Results:** A total of 1,201 mutations spanning 91 genes were detected from 234 patients, resulting in a mutation detection rate of 80.7%. TP53 (64.1%) was the gene with highest mutation frequency, followed by PIK3CA (31%), PTEN (11%), and RB1 (10%). Copy number amplifications (CNAs) in MYC (14.1%), FGFR1 (13.3%), CCND1 (6.6%), FGF3 (6.6%), FGF4 (6.2%) and FGF19 (6.2%) were also detected from our cohort. TP53 mutations were significantly more frequent among triple negative BC (TNBC), HR−/HER2+, and HR+/HER2+ BC, while less common in HR+/HER2– (*P* < 0.01). Meanwhile, PIK3CA mutations were significantly more frequent among HR+/HER2+, HR+/HER2–, and HR−/HER2+ BC, while less common in TNBC (*P* < 0.01). Pathogenic or likely pathogenic BRCA1/2 germline mutations were detected in 5.9% of the cohort and 4.4% in TNBC subgroup. Maximum allelic fraction (maxAF) of TP53, RB1, and PIK3CA mutations were associated with multiple organ metastasis. Patients with PIK3CA, PTEN, and RB1 mutation were more likely to have liver metastasis (*P* < 0.02). Compared with MSKCC and PCAWG dataset, Chinese patients had observably difference in genetic variation rates in different molecular subtypes (TNBC: TP53 73.0 vs. 91.5%, *P* < 0.001; PIK3CA 21.2 vs. 13.2%, *P* = 0.061; HR+/HER2−: FGFR1 3.3 vs. 0.7%, *P* = 0.035; TP 53 46.2 vs. 27.7%, *P* < 0.001; RB1 6.6 vs. 2.7%, *P* = 0.046; CDKN2A 7.7 vs. 1.0%, *P* < 0.001; PIK3CA 30.8 vs. 44.2%, *P* = 0.012; CDH1 1.1 vs. 18.2%, *P* < 0.001; GATA3 7.7 vs. 17.2%, *P* = 0.02).

**Conclusions:** A distinct gene mutation profile was elucidated in Chinese women with metastatic BC, justifying further research. Liquid biopsy provides a quick, real-time, and minimally invasive tool for future clinical trial and routine practice.

## Introduction

Breast cancer is a disease with clinical and molecular heterogeneity (1, 2). Based on their distinct molecular expression profiles, it has been classified into four molecular subtypes into Luminal-A, Luminal-B, HER2–positive and basal-like (3). This molecular subclassification is evaluated through immunohistochemical (IHC) analysis of the expression of biomarkers including estrogen receptor (ER), progesterone receptor (PR), human epidermal growth factor receptor 2 (HER2) and antigen Ki-67 (3, 4). Due to their intrinsic molecular heterogeneity, response to treatment varies among different molecular subtype; hence, proper molecular subtyping is necessary to guide optimal treatment decisions and evaluate the prognostic outcome of the patients (4–6). High-throughput sequencing technologies and bioinformatics tools accelerated the comprehensive understanding of the molecular heterogeneity of breast cancer paving the way in identifying more targetable mutations and personalizing treatment strategy of patients (7). Growing efforts have been invested in elucidating the mutation profile of breast tumors of various histology and stages to identify oncogenic drivers that could potentially be targeted by therapy and other molecular factors that could affect treatment response to certain therapy or markers that could predict survival outcomes of patients (1, 8–10).

Recent studies have elucidated the comprehensive mutation profile and identified frequently mutated genes in Chinese women with treatment naïve early-stage breast tumors (10). However, as compared with early-stage breast cancer, metastatic disease has already spread to other organs and would have more clinically and molecularly complex features. This is supported by recent study which revealed significant single nucleotide variation between primary and metastatic breast cancer (11–13). Numerous studies interrogating the comprehensive molecular profile of metastatic breast tumors are available (11–14); however, these studies involve Caucasian patients. Meanwhile, such comprehensive investigation has not been conducted among the Chinese population.

In our study, we aimed to elucidate the comprehensive mutation profile and identify frequently-altered genes among Chinese women with metastatic breast cancer.

## Patients and Methods

### Patients

Female patients with metastatic breast cancer diagnosed at Fudan University Shanghai Cancer Center (FUSCC) from January 2017 to April 2019 were included in the study. Clinical and pathological information were obtained from each patient including age, pathological type, ER, PR, HER2, and Ki67 status, number and details of metastatic sites, and details of prior treatment as well as clinical course. The expression of ER, PR and HER2 for each patient was analyzed by IHC staining at the Department of Pathology of the FUSCC. ER or PR positivity was defined as strong staining in more than 1% of the tumor nuclei, according to the 2010 guidelines of the American Society of Clinical Oncology (ASCO)/College of American Pathologists (CAP) (15). HR positivity was described as either ER-positive (ER+) or PR positive (PR+), while HR-negative (HR-) status was defined when both ER and PR expression were negative. HER2 status required further confirmation by fluorescence *in situ* hybridization (FISH) when expression status evaluated by IHC was 2+, according to the 2013 ASCO/CAP guidelines (16). The Ethics Committee of FUSCC has granted approval for this study (Approval number: 1705172-9). Written informed consent was provided by each patient.

### Circulating DNA Extraction

After collection, peripheral blood samples were processed within 72 h to separate the plasma from the peripheral blood cells, and transferred to fresh tubes for storage at −80°C until DNA isolation. DNA isolation and subsequent sequencing procedures were performed in the laboratory of Burning Rock Biotech (Guangzhou, China) accredited and certified by the CAP and Clinical Laboratory Improvement Amendments (CLIA). Circulating cell-free DNA (cfDNA) were extracted using QIAamp Circulating Nucleic Acid Kits (Qiagen, Hilden, Germany) from 0.5–2.0 mL of the plasma samples. Genomic DNA (gDNA) used as normal control were extracted from white blood cells (WBCs) by QIAamp DNA Blood Mini Kit (Qiagen, Hilden, Germany). Qubit fluorometer with the dsDNA high-sensitivity assay kit (Life Technologies, Carlsbad, CA, USA) was used to measure DNA quality following the manufacturer's instructions.

### Next-Generation Sequencing (NGS) Library Preparation, Capture-Based Targeted DNA Sequencing and Sequence Data Analysis

NGS library was constructed for the DNA isolated from plasma and white blood cells according to optimized protocol. A minimum of 50 ng of DNA is required for NGS library construction. Target capture was performed using commercially-available panels consisting of 108 breast cancer-related genes (PurePlasma) and 520 cancer-related genes (OncoScreen Plus), spanning 0.249 megabases (Mb) and 1.64 Mb of the human genome, respectively (Burning Rock Biotech, Guangzhou, China). The genes included in the panel are listed in Tables S1, S2, respectively. Indexed samples were sequenced on Nextseq500 sequencer (Illumina, Inc., US) with paired-end reads achieving target coverage of 10,000X for plasma samples. Sequencing data were analyzed using proprietary computational algorithms optimized for somatic variant calling as described previously (17, 18). Variants with population frequency over 0.1% in the ExAC, 1000 Genomes, dbSNP or ESP6500SI-V2 databases were grouped as single nucleotide polymorphisms and excluded from further analysis. Variants detected from the patient's own WBCs were filtered out to retain only the somatic variants. Only the variants with pathogenic/likely pathogenic classification based from ClinVar and other similar databases identified from the WBCs were flagged for reporting of incidental findings. Copy number variations (CNV) were analyzed based on the depth of coverage data of capture intervals. Coverage data were corrected against sequencing bias resulting from GC content and probe design. The average coverage of all captured regions was used to normalize the coverage of different samples to comparable scales. CNV was calculated based on the ratio between the depth of coverage in patient samples and average coverage of an adequate number of samples without copy number variations (n>50) as references per capture interval. CNV is called if the coverage data of the gene region was quantitatively and statistically significant from its reference control. The limit of detection for CNVs is 1.5 and 2.64 for deletions and amplifications, respectively.

### Statistical Analysis

Categorical data were described by frequency and percentage. Quantitative variables were expressed as means ± SEM. Fisher's exact or Chi-square test was performed to compare categorical variables. The Student *t*-test was used for analyzing quantitative data between two groups, and one-way ANOVA was used for comparisons of more than two groups. All the data were analyzed via R statistics package (R version 3.5.3; R: The R-Project for Statistical Computing, Vienna, Austria). All statistical tests were two-sided, and *P*-value of < 0.05 were considered significant.

## Results

### Patient Characteristics

Between January 2017 and April 2019, 290 women diagnosed with metastatic breast cancer at FUSCC were included in this retrospective cohort. The median age of the cohort was 49.5 years, with a range of 22 to 77 years. Among the patients with menopausal status data, 61.1% (160/262) of them were menopausal. Of the 290 patients, 47.2% (137/290) had TNBC, 31.3% (91/290) had HR+/HER2−, 6.9% (20/290) had HR+/HER2+, and 12.4% (36/290) had HR−/HER2+ breast cancer. The remaining 2.1% (6/290) had unknown molecular subtype. Visceral metastasis was found in 72.4% (210/290), 42.8% (124/290) presented with bone metastasis and 5.5% (16/290) with brain metastasis. About 15.5% (45/290) of the patients had already received 4 or more lines of treatment to manage their metastatic disease. The detailed clinicopathological features of the cohort were summarized in Table 1.

**Table 1:** Clinicopathological features of the cohort.

**Clinicopathological features**	***n* = 290 *n* (%)**
Age (median, range)	49.5 (22–77)
Menopausal status (*n* = 262)	
Pre-menopausal	102 (38.9%)
Menopausal	160 (61.1%)
Molecular subtype	
TNBC	137 (47.2%)
HR+/HER2−	91 (31.4%)
HR+/HER2+	20 (6.9%)
HR−/HER2+	36 (12.4%)
Unknown	6 (2.1%)
Metastatic site	
Breast	6 (2.1%)
Bone	124 (42.8%)
Liver	114 (39.3%)
Lung	134 (46.2%)
Brain	16 (5.5%)
Prior treatment lines	
0	108 (37.2%)
1	53 (18.3%)
2	39 (13.4%)
3	38 (13.1%)
4	16 (5.5%)
5–9	29 (10%)
Unknown	7 (2.5%)

### Mutational Landscape of Chinese Metastatic Breast Cancer

**Figure 1:**
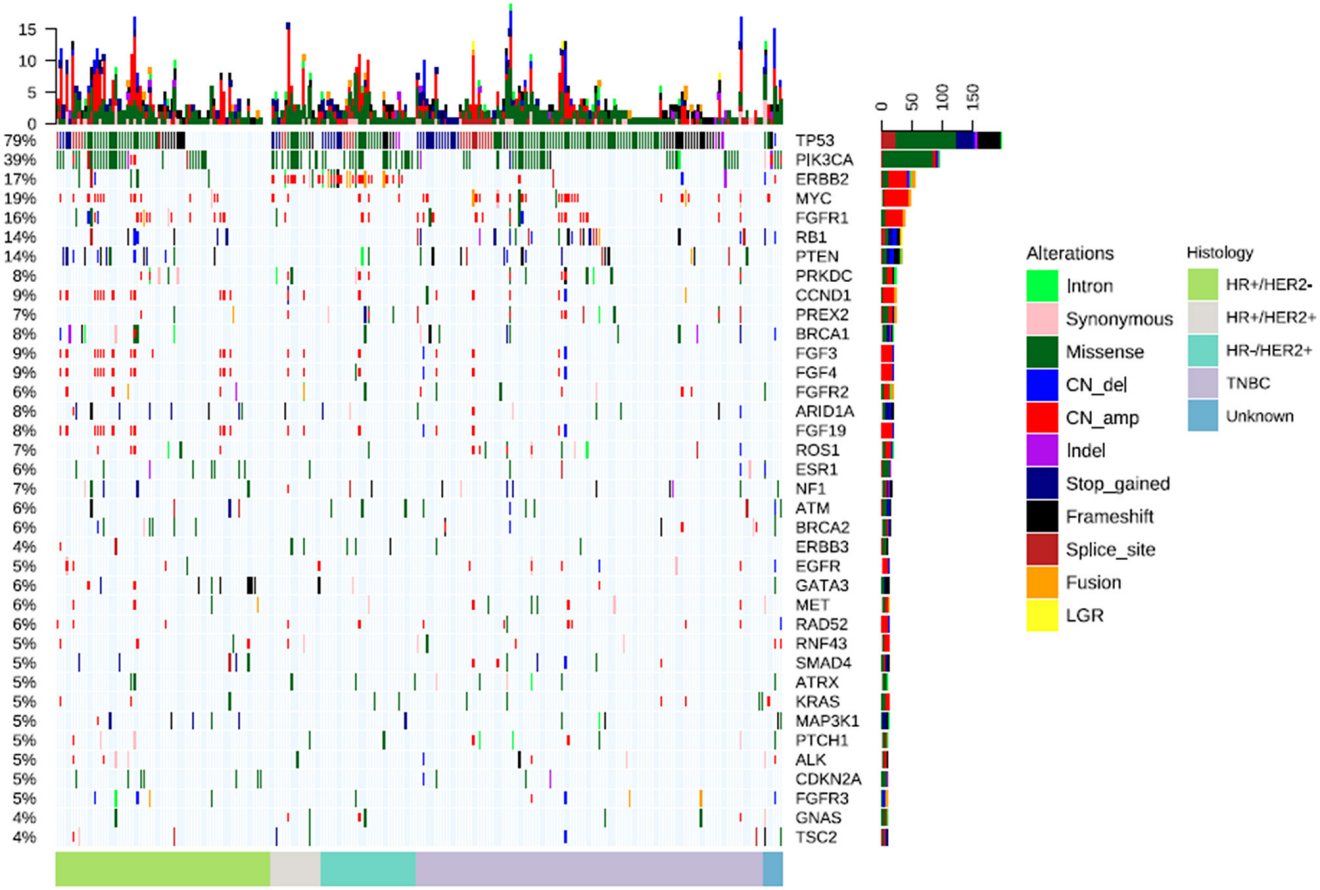
CtDNA mutation profile of the cohort. The bars located at the bottom of the oncoprint denote molecular histology subtypes. Each column represents a patient and each row represents a gene. Values on the left represent the mutation rate of each gene. Values on the right indicate the genes. Top plot represents the overall number of mutations a patient carried. Different colors denote different types of mutation.

To elucidate the comprehensive mutational landscape of Chinese metastatic breast tumors, plasma samples obtained from 290 patients were sequenced using a targeted panel with either 108 (*n* = 164) or 520 (*n* = 126) cancer-related genes. A total of 91 genes from the 108-gene panel were also included in the 520-gene panel; hence, only the mutation profiles for the 91 genes were analyzed for all patients. A total of 1,201 mutations were detected, including 517 single nucleotide variations (SNVs), 123 insertion-deletion variants (Indels), 23 fusions, 61 splice-site variants, 76 nonsense variants, 401 copy number amplifications (CNA), and 156 copy number deletions, spanning 91 genes from 234 patients, revealing a mutation detection rate of 80.7% (234/290). The average allelic fraction detected from the ctDNA was 4.52%, ranging from 0–88.20%. TP53, detected from 64.1% of the patients, was the most commonly mutated gene in our cohort. PIK3CA (31%), PTEN (11%), and RB1 (10%) were also identified to be mutated in more than 10% of the cohort. In addition, CNAs, including MYC (14.1%), FGFR1 (13.3%), CCND1 (6.6%), FGF3 (6.6%), FGF4 (6.2%), and FGF19 (6.2%) were commonly detected in our cohort. Figure 1 illustrates the distribution of the genomic alterations detected from our cohort.

### Mutation Landscape According to Molecular Subtype

We further analyzed the distribution of mutations according to the 4 molecular subtypes. A total of 92 mutations were detected from HR+/HER2+, 398 mutations from HR+/HER2−, 498 mutations from TNBC, and 144 mutations from HR−/HER2+ tumors, revealing mutation detections rates of 80.0% (16/20), 76.9% (70/91), 83.9% (115/137), and 86.1% (31/36), respectively. Overall mutation rates and types were not statistically different among the breast cancer molecular subtypes (*P* = 0.21). The distribution of mutation types according to the molecular subtype are summarized in Table S3.

**Figure 2:**
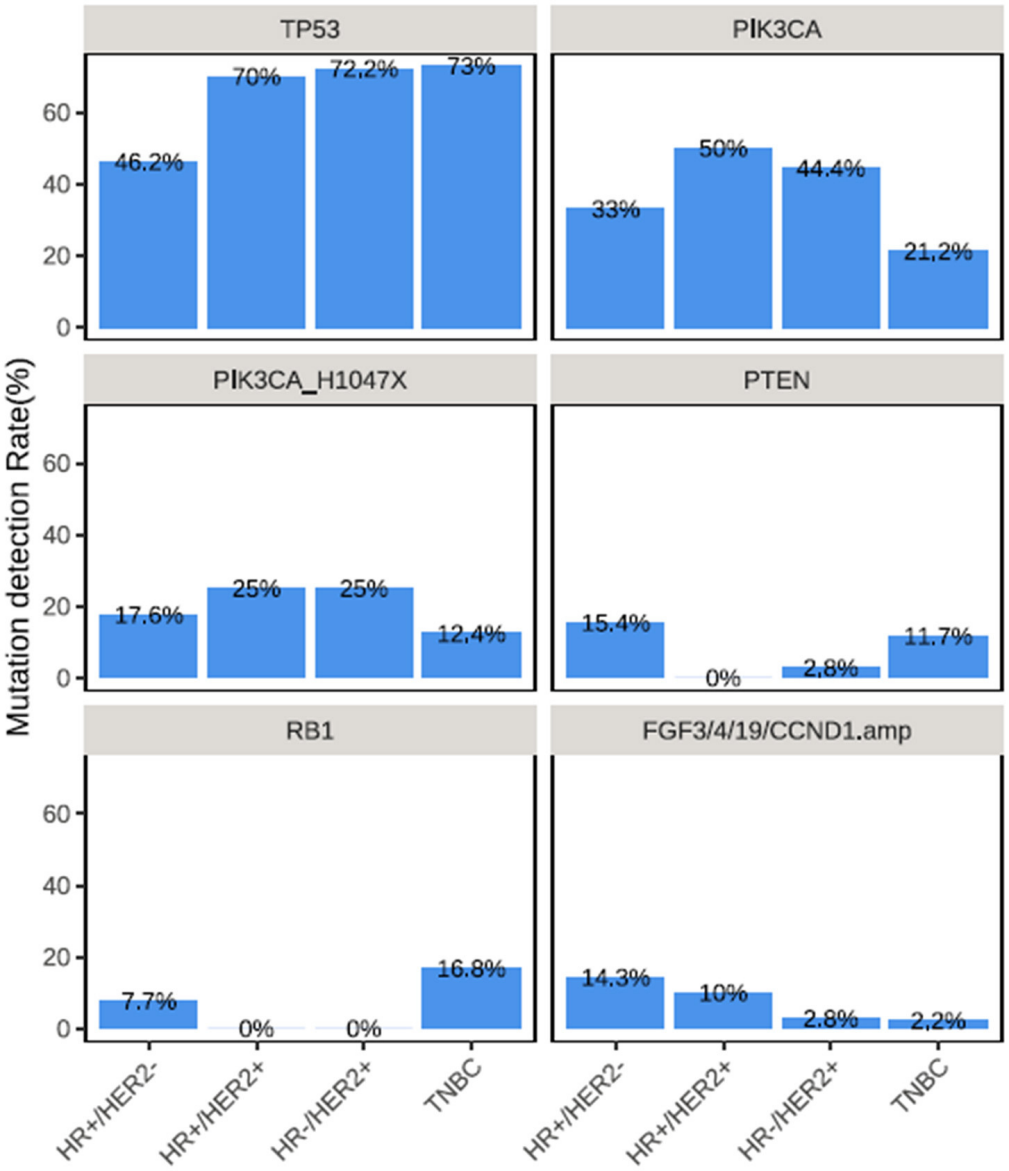
Distinct mutation rate across breast cancer molecular subtypes. Mutation rates of various genes across the molecular subtypes in TP53, PIK3CA, PIK3CA H1047X, PTEN, RB1, and collectively in FGF3, FGF4, FGF19, and CCND1.

The frequently altered genes across all 4 molecular subtypes were described in Figure 2. The TP53 detection rates in TNBC (73.0%, 100/137) and HR−/HER2+ tumors (72.2%, 26/36) and HR+/HER2+ (70.0%, 14/20) were significantly higher than in HR+/HER2− (46.2%, 42/91) breast cancer (*P* < 0.01). Meanwhile, PIK3CA mutations were more frequently detected from HR+/HER2+, HR−/HER2+ and HR+/HER2− breast cancer and less common in TNBC (*P* < 0.01), with detection rates of 50.0% (10/20), 44.4% (16/36), 33.0% (30/91), and 21.2% (29/137), respectively (Figure 2). The most common PIK3CA mutation was H1047X, which was detected from 25.0% (5/20), 17.6% (16/91), 25.0% (9/36), and 12.4% (17/137) of the patients with HR+/HER2+, HR+/HER2−, HR−/HER2+, and TNBC, respectively.

PTEN mutations were more frequently detected among patients with HR+/HER2− (15.4%, 14/91) and TNBC (11.7%, 16/137) (*P* = 0.035; Figure 2). RB1 mutations were predominantly detected among patients with TNBC (16.8%, 23/137, *P* = 0.003), while RB1 mutations were not detected among patients with HR+/HER2+ and HR−/HER2+ tumors (Figure 2). CNAs in CCND1, FGF3, FGF4, and FGF19, which are all located in the q-arm of chromosome 11 were more likely to be detected from patients with HR+/HER2+ and HR+/HER2− breast cancer (*P* < 0.001) (Figure 2).

### Somatic and Germline BRCA1/2 Mutations Detected From the Cohort

Pathogenic or likely pathogenic mutations in BRCA1/2 genes were identified in 5.9% (17/290) from our cohort. Of which, 6 mutations were found to be somatic; while 11 were confirmed to be germline mutations. Specifically, germline BRCA1 and BRCA2 mutations were detected in 7 and 4 patients, respectively; while somatic BRCA1 and BRCA2 mutations were detected in 4 and 2 patients, respectively. Germline and somatic BRCA1/2 mutations were detected from 4.4% (6/137) and 3.7% (5/137) patients with TNBC, respectively. Meanwhile, germline BRCA1/2 mutations were detected from5.5% (5/91) of the patients with HR+/HER2− tumors. Interestingly, no germline or somatic BRCA1/2 was detected among patients with HR+/HER2+ and HR−/HER2+ patients. The distribution of pathogenic/likely pathogenic germline and somatic BRCA1/2 mutations detected from the cohort was summarized in Table S4.

### Comparison of the Mutation Profile Between Our Cohort and Two Publicly-Available Datasets of Metastatic Breast Cancer Patients

To further explore the differences in the frequency of mutation in metastatic breast tumors between Chinese and Caucasian women, we compared the data from our cohort with publicly-available dataset from the Memorial Sloan Kettering Cancer Center (MSKCC) and Pan-cancer Analysis of Whole Genomes (PCAWG), respectively consisting of 1,855 and 447 Caucasian advanced breast cancer patients with known clinical information. Among the patients with metastatic breast cancer included in the MSKCC dataset, 77.0% (1,429/1,855) had HR+/HER2−, 9.2% (170/1,855) had HR+/HER2+, 4.4% (82/1,855) had HR−/HER2+, and 9.4% (174/1,855) had TNBC. Meanwhile, among the patients with metastatic breast cancer enrolled in the PCAWG cohort, 67.3% (301/447) had HR+/HER2−, 11.6% (52/447) had HR+/HER2+, 7.4% (33/447) had HR−/HER2+, and 13.6% (61/447) had TNBC. MSKCC cohort was sequenced using targeted sequencing panel which covered either 341 or 468 genes (MSK-IMPACT), while the PCAWG cohort was sequenced using whole genome sequencing which only attained average sequencing depth of 106X for tumor samples. This ensures that the analysis of mutation rates in SNVs and Indels among our cohort and MSKCC and PCAWG would provide meaningful data, whereas mutation rates in CNVs would be limited by the choice of sample from our cohort, the exclusion of the genes in the MSKCC cohort, or the limited sequencing coverage in PCAWG cohort.

**Figure 3:**
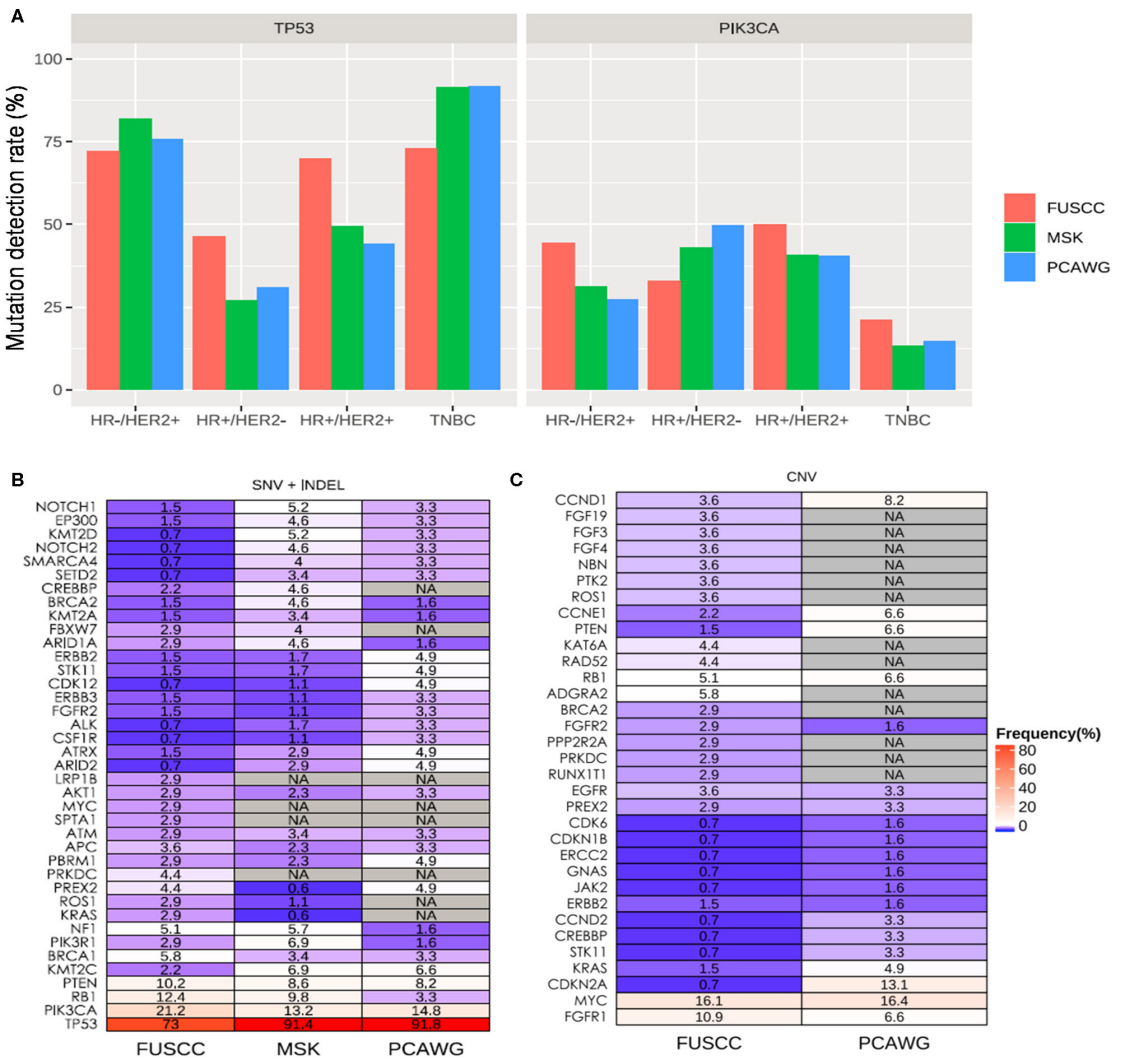
Mutation spectrum between Chinese and Caucasian breast cancer patients. **(A)** TP53 mutation is significantly higher in Chinese patients with HR+/HER2− and HR+/HER2+ compared to either MSKCC and PCAWG, Significantly higher PIK3CA mutations are found in patients with HR+/HER2− from our cohort. Blue bars represent the data from our cohort. Red bars represent the data from the MSKCC dataset, while green bars represent the data from the PCAWG dataset. The mutated genes including SNV and INDEL **(B)** and CNV **(C)** types in TNBC patients among our Chinese cohort (FUSCC) and Caucasian patients with metastatic breast tumors from publicly-available datasets from Memorial Sloan Kettering Cancer Center (MSK) and Pan-Cancer Analysis of Whole Genomes (PCAWG). NA denotes not detected.

Upon inspection of the mutation profiles among the three cohorts, distinct differences can be observed in mutation rates of genes for SNVs and Indel; however, no statistical difference was found. Comparison of the TP53 and PIK3CA mutation rates revealed that our cohort had no significant difference as compared to either MSKCC (TP53: 65.4 vs. 62.5%; *P* > 0.05; PIK3CA: 37.2 vs. 32.0%; *P* > 0.05) or PCAWG (TP53: 65.4 vs. 60.7%; *P* >.05; PIK3CA: 37.2 vs. 33.1%; *P* > 0.05) cohorts across all molecular subtypes. However, our TNBC cohort had significantly lower mutation frequency in TP53 than both MSKCC (73.0 vs. 91.4%; *P* < 0.001) and PCAWG (73.0 vs. 91.8%; *P* = 0.005) cohorts (Figure 3A). We found Chinese metastatic TNBC patients had higher but not significantly mutation rates of RB1, PIK3CA, and PTEN mutations compared with MSKCC dataset (RB1:12.4 vs. 9.8%, *P* = 0.47; PIK3CA: 21.2 vs. 13.2%, *P* = 0.06; PTEN: 10.2 vs. 6.9%, *P* = 0.78), or PCAWG dataset (RB1: 12.4 vs. 3.3%, *P* = 0.06; PIK3CA: 21.2 vs. 14.8%, *P* = 0.33; PTEN: 10.2 vs. 8.2, *P* = 0.85) (Figure 3B). There was no difference in BRCA 1 mutation between our cohort and TNBC cohort from the MSKCC (5.8 vs. 3.4%, *P* > 0.05) and PCAWG dataset (5.8 vs. 3.3%, *P* > 0.05) (Figure 3B).

Since MSKCC cohort did not include CNV analysis of the same genes as our panel, their dataset was not included for the CNV analysis. Meanwhile, the whole genome sequencing data from PCAWG cohort might lack adequate sequencing coverage in certain regions of the genes covered by our panel. In addition, ctDNA also has limited sensitivity for CNV analysis. A comparison of CNVs between our TNBC cohort and that of PWACG demonstrates distinct differences in mutation rates (Figure 3C); however, statistical tests were not performed due to inherent bias resulting from depth of coverage limitations and sample type differences.

When compared with MSCKCC and PCAWG combined data, we found additional genetic variations in Chinese metastatic patients besides the rates of TP and PIK3CA consistently with the results above (TP3:73.0 vs. 91.5%, *P* < 0.001; PIK3CA: 21.2 vs. 13.6%, *P* = 0.061). Chinese metastatic HR+/HER2− patients had significantly higher rates of FGFR1 (3.3 vs. 0.7%, *P* = 0.035), TP 53 (46.2 vs. 27.7%, *P* < 0.001), RB1 (6.6 vs. 2.7%, *P* = 0.046), and CDKN2A (7.7 vs. 1.0%, *P* < 0.001), and had markedly lower frequencies of PIK3CA (30.8 vs. 44.2%, *P* = 0.012), CDH1 (1.1 vs. 18.2%, *P* < 0.001) and GATA3 (7.7 vs. 17.2%, *P* = 0.02).

### Correlation of Clinical and Molecular Features

**Figure 4:**
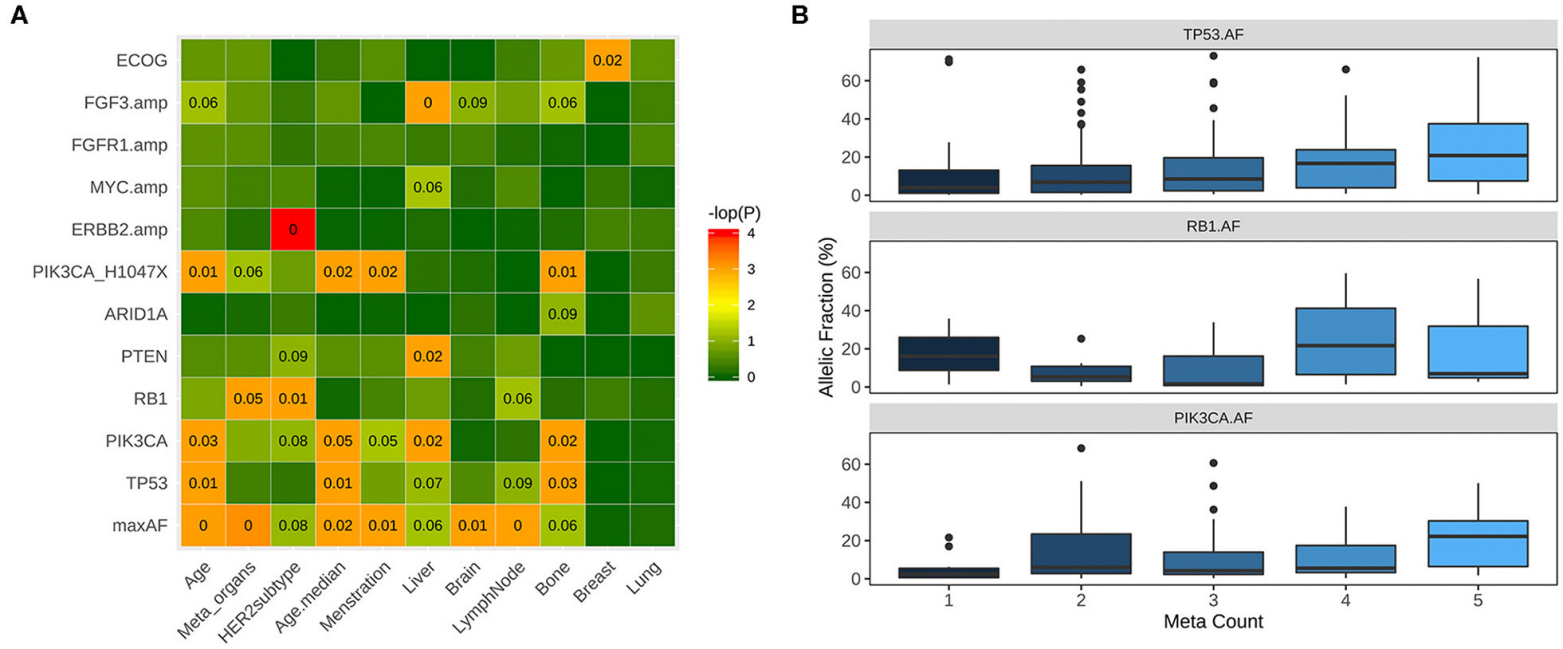
Correlation between clinical and molecular features. **(A)** PIK3CA-mutant patients were significantly more likely to harbor metastasis in the bone, liver and other organs including liver, lung, and brain. Patients with mutations in PTEN and loss of function (LOF) mutations in ARID1A have significantly higher likelihood of liver and bone metastasis, respectively. **(B)** Patients with more metastasis have significantly higher allelic fractions of mutations of PIK3CA, TP53 and RB1. Box plot illustrating the higher mutation allelic fractions of patients with 1, 2, 3, 4, or 5 metastatic sites (meta count).

We further evaluated the correlation between clinicopathological features and molecular features of the cohort. Since metastasis might be influenced by other clinical factors, we have adjusted the *P*-values with histology and ER status of the patients as reflected by the adjusted *P*-values (adjP). PIK3CA-mutant patients were more likely to harbor metastasis to multiple organs including brain, lung, and liver (94.3 vs. 70.1%, *P* = 0.02, adj *P* = 0.0047), particularly to the liver and bone (53.9 vs. 37.4%, *P* = 0.01, adj *P* = 0.022, 55.1 vs. 38.8%, *P* = 0.01, adj *P* = 0.024, respectively, Figure 4A and Figure S1A). Moreover, patients harbored PIK3CA H1047X mutation were significantly associated with bone metastasis (61.2 vs. 40.6%, *P* = 0.004, adj *P* = 0.0025, Figure 4A and Figure S1B). In addition, patients with PTEN and RB1 mutations were also more likely to harbor liver metastasis (68.8 vs. 21.8%, *P* = 0.003, adj *P* = 0.0014, 56.5 vs. 21.4%, *P* = 0.002, adj *P* = 0.024; respectively, Figure 4A and Figure S1C). Patients with loss of function mutations of ARID1A (72.7 vs. 143.6%, *P* = 0.02, adj *P* = 0.13, Figure 4A and Figure 1D) were more likely to develop bone metastasis. Moreover, patients with more metastasis had significantly higher maximum allelic fraction (maxAF) of mutations in TP53 (*P* = 0.01), RB1 (*P* = 0.03), and PIK3CA (*P* = 0.05) (Figure 4B). MaxAF was defined as the highest allelic fraction among all the somatic mutations detected from the panel used regardless of gene. Meanwhile, metastatic count was defined as the total number of metastatic organ sites of each patient.

## Discussion

In recent years, extensive efforts have been invested in exploring the mutation landscape of breast cancer to understand its genomic complexity (1, 8, 10, 11, 13, 14). However, gaps in existing knowledge still remain. To the best of our knowledge, our study is the first to elucidate the comprehensive molecular profile of metastatic breast tumors in the Chinese population.

Our retrospective study elucidated the mutation landscape of metastatic breast tumors in Chinese women. In our cohort, the most commonly mutated genes included TP53, PIK3CA, RB1, and PTEN. Meanwhile, CNAs were frequently identified in MYC, FGFR1, ERBB2, CCND1, FGF3, FGF4, and FGF19. Among these genes, at least one mutation was detected from 74.8% (217/290) of our cohort, revealing the important role of these genes in the development of metastatic breast cancer. As compared to previous reports on Chinese early-stage breast tumors, the mutation rate of PIK3CA was lower (44 vs. 39% from our cohort) and TP53 was higher (45 vs. 79% from our cohort) in metastatic BC (10), and the gene amplification of FGFR1 was only found in metastatic TNBC (0 vs. 13.3% from our cohort) (19), suggesting the molecular distinction between early and metastatic breast tumors. This finding contributes an incremental step in understanding the molecular complexity of metastatic breast tumors.

HR+ breast tumors regardless of HER2 status (HR+/HER2+ and HR+/HER2−) were more likely to harbor CNAs in CCND1, FGF3, FGF4, and FGF19, which colocalizes in chromosome 11q13.3. CNAs involving the chromosome 11q13, particularly CCND1, have been identified in patients with ER-positive tumors and are associated with poor long-term survival and treatment failure (20–23). CCND1 amplification is commonly mutated in various solid tumors and should be highly sensitive to cyclin-dependent kinase (CDK) 4/6 inhibition (24). However, both CCND1 amplification and PIK3CA mutations were not predictive of therapeutic benefit from CDK4/6 inhibitor palbociclib in patients with HR-positive metastatic breast cancer (25).

Our findings have also revealed the correlation between harboring mutations in PIK3CA such as PIK3CA H1047X, and the presence of various metastases particularly in the bone, suggesting the role of PIK3CA in metastatic development. PIK3CA pathway is one of the most frequently deregulated pathways in breast cancer and has been implicated in breast tumor development, progression and therapeutic resistance (26). Somatic mutations in PIK3CA have been demonstrated to be associated with HR-positive (either ER-positive or PR-positive)/HER2−negative breast tumors (26–29). An increase in PIK3CA mutations have been observed in relapsed breast tumors as compared to primary breast tumors (30). In addition, PIK3CA mutations were more likely to be observed among patients with HER2-positve breast tumors who have liver metastases (29, 31). The frequency of PIK3CA mutations in breast cancer has also attracted attention as a potential drug target (32). Consistent with these studies, our findings raise the clinical value of PIK3CA mutations as prognostic biomarker. Several selective inhibitors have been developed to target PIK3CA and are currently being investigated (32). The promising results from the SOLAR-1 clinical trial have resulted in the recent approval of alpelisib in the treatment of PI3KCA-mutant, HR-positive advanced breast cancer (33).

The Cancer Genome Atlas (TCGA) project, a joint effort between the National Cancer Institute and the National Human Genome Research Institute, has comprehensively profiled the genome of more than 11,000 patients with 31 solid cancer types (1, 34). The TCGA has contributed vastly in our current understanding of the molecular heterogeneity of primary tumors in various cancer types. Other pan-cancer sequencing efforts including the PCAWG project have extended our knowledge regarding the mutational landscape of metastatic cancer in 20 cancer types from 2,399 patients (13). In addition, numerous efforts have also elucidated cancer-specific comprehensive mutational landscape which provided valuable insights in the mutational heterogeneity of patients with advanced breast cancer (11, 12, 14). However, the patients included in these large-scale studies were predominantly Caucasians with an underrepresentation of cancer patients of other ethnic backgrounds including Asians (34). The molecular diversity associated with oncogenesis between Caucasians and Asians have been established by the identification of EGFR sensitizing mutations in non-small cell lung cancer, wherein EGFR-mutant tumors are more prevalent in Asians than Caucasians (50 vs. 10%) (34–36). Meanwhile, in breast cancer, only the study by Liao and colleagues has comprehensively profiled early-stage breast tumors of Chinese patients and found ethnic distinction between their cohort and the TCGA (10). Consistently, our findings also demonstrate that Chinese metastatic breast tumors have a distinct molecular profile as compared to Caucasian metastatic breast tumors from MSKCC and PCAWG datasets. Chinese TNBC patients harbored a significantly lower frequency of TP53 mutations than Caucasian TNBC patients (MSKCC: 73.0 vs. 91.4%, *P* < 0.001; PCAWG: 73.0 vs. 91.8%, *P* = 0.005). Moreover, another recent study on ctDNA molecular profiling in Caucasian patients with metastatic breast cancer has reported a mutation rate of 52% and 40% for TP53 and PIK3CA, respectively (37). This distinct mutation profile might suggest that drug response might also be different among Chinese and Caucasian patients, indicating the need to reevaluate treatment strategies in the Chinese population.

Plasma, being less invasive as compared to tissue biopsy, is now commonly utilized in the clinical setting as an alternative source of tumor DNA for mutation profiling (38, 39). The ctDNA concentration present in the circulation is directly related to the tumor burden of a patient; hence, plasma samples could serve as a better representation of the tumor heterogeneity in metastatic disease (38, 39). Interestingly, a recent study has explored the potential of ctDNA as a prognostic tool in metastatic breast cancer (37). However, the use of ctDNA-based molecular profiling of metastatic breast cancer in clinical settings is still limited. Based on our results, the mutation detection rate from plasma ctDNA was 82.1%. This high mutation detection rate indicates that plasma ctDNA-based mutational profiling is also applicable in metastatic breast cancer patients. However, since ctDNA is considered to be released by apoptotic or necrotic tumor cells or directly secreted by the tumor cells as exosomes, ctDNA is comprised of shorter fragments of DNA (38, 39), which makes it technically limiting to detect copy number variations. Due to this limitation, ERBB2 amplifications were only detected from 54.1% (20/37) of the patients with HER2-positive tumors in our cohort, which indicates a concordance of only 54.1% between genomic profiling-based CNA and immunohistochemistry-based HER2 expression. Conversely, since the molecular profile of metastatic breast tumors would differ from the primary breast tumor, ctDNA could reflect the heterogeneity of metastatic disease. Numerous studies have demonstrated the discordance in ER, PR, and HER2 receptor status (27, 40, 41) and other biomarkers, including PTEN and PIK3CA (27), between the primary and metastatic tumors. This discordance could significantly influence treatment response and patient prognosis, indicating the need to evaluate these biomarkers not only from primary tumors at baseline, but also at progression and evaluation of tissues from metastatic sites (27). Nonetheless, ctDNA profiling could still provide mutation landscape for therapeutic guidance.

Due to the retrospective nature of our study, a few limitations are associated with this study. First, the lack of tissue samples for comparison of the molecular profile obtained from the plasma samples. Since the fragment size of ctDNA derived from plasma samples are smaller as compared to the tumor DNA purified from tissue samples, the detection of copy number variants and structural variants are limited and needs further verification. Second, the baseline collection of blood samples was not performed at the same time for all the patients. Most of our patients have already undergone numerous lines of treatment; hence, we have also evaluated the influence of regimens on the mutation profile of the patients. The results showed that both ctDNA allelic fraction and mutation profiles as a whole were not affected by treatment lines (data not shown). Third, since the overall survival data for our cohort is not yet mature, the prognostic values of frequently altered genes, such as TP53, PIK3CA, and MYC need further confirmation. Fourth, since our study was only conducted in a single center with a limited number of patients, further investigation is necessary to validate our findings in a prospective study with a larger cohort that could be achieved with a multi-center collaboration.

## Conclusion

In conclusion, we revealed the distinct mutation landscape of Chinese metastatic breast cancer, which is significantly different from Caucasian tumors and early-stage tumors. Our findings also demonstrate that ctDNA mutation profiling is a tool that could simultaneously assess the molecular landscape and elucidate the molecular features of the disease. These findings could pave the way in improving treatment planning for patients based on the mutation profile of their respective tumors.

## Data Availability Statement

The datasets presented in this study can be found in online repositories. The names of the repository/repositories and accession number(s) can be found below: NCBI under BioProject PRJNA616082.

## Ethics Statement

The studies involving human participants were reviewed and approved by Ethics Committee of Fudan University Shanghai Cancer Center. The patients/participants provided their written informed consent to participate in this study.

## Author Contributions

Conception and design: ZT and XH. Acquisition of data (acquired and managed patients, provided facilities, etc.): TL, ZF, CL, MZ, YS, JZ, BW, LW, and JC. Analysis and interpretation of data (e.g., statistical analysis, biostatistics, *in silico* analysis): ZT, BL, and AL. Writing, review, and/or revision of the manuscript and approval of final manuscript: All authors. All authors contributed to the article and approved the submitted version.

## Conflict of Interest

AL and BL were employed by the company Burning Rock Biotech. The remaining authors declare that the research was conducted in the absence of any commercial or financial relationships that could be construed as a potential conflict of interest.

## Abbreviations

cfDNA: cell-free DNA
CNA: copy number amplifications
CNV: copy number variations
ctDNA: circulating tumor DNA
ER: estrogen receptor
FUSCC: Fudan University Shanghai Cancer Center
HER2: human epidermal growth factor receptor 2
HR: hormone receptor
IHC: immunohistochemistry
maxAF: maximum allelic fraction
MSKCC: Memorial Sloan Kettering Cancer Center
NGS: next-generation sequencing
PCAWG: pan-cancer analysis of whole genomes
PR: progesterone receptor
SNV: single nucleotide variants
TCGA: The Cancer Genome Atlas
TNBC: triple negative breast cancer.
