# Supplementary material for: Characterizations of Cancer Gene Mutations in Chinese Metastatic Breast Cancer Patients
Source: Front Oncol. 2020 Jun 30;10:1023. doi: 10.3389/fonc.2020.01023 (PMC7338574; doi:10.3389/fonc.2020.01023)
Supplement: Supplementary file 1 [file Data_Sheet_1.docx]

**Table S1.** The 108 cancer-related genes included in the PurePlasma panel

| AKT1 | CBL | EGFR | HDAC2 | MYC | PIK3R1 | RUNX1 | TTK |
| --- | --- | --- | --- | --- | --- | --- | --- |
| ALK | CCND1 | ERBB2 | HRAS | NEB | PPP2R1A | SETD2 | VHL |
| APC | CDH1 | ERBB3 | IDH1 | NEFH | PREX2 | SF3B1 | NRG1 |
| AQP7 | CDK4 | ERBB4 | IDH2 | NF1 | PRKDC | SMAD4 |  |
| ARID1A | CDK6 | ESR1 | JAK1 | NF2 | PTCH1 | SMO |  |
| ATM | CDKN2A | FBXW7 | KDM5A | NFE2L2 | PTEN | SOX2 |  |
| ATP2B3 | CHEK1 | FGF19 | KIF6 | NOTCH2 | RAD50 | SPOP |  |
| ATR | CHEK2 | FGF3 | KIT | NOTCH4 | RAD51 | STK11 |  |
| ATRX | CSMD1 | FGF4 | KRAS | NRAS | RAD52 | SYNE1 |  |
| BCL2L11 | CSMD3 | FGFR1 | MAP3K1 | NTRK1 | RB1 | TCF12 |  |
| BRAF | CTNNA2 | FGFR2 | MED12 | OMG | RET | TERT |  |
| BRCA1 | CTNNB1 | FGFR3 | MET | PBRM1 | RHOA | TGFBR2 |  |
| BRCA2 | CYP4F3 | FLT3 | MSH6 | PCDH11X | RIMS2 | TP53 |  |
| CACNA1C | DDR2 | GATA3 | MTOR | PDGFRA | RNF43 | TSC1 |  |
| CBFB | DGKB | GNAS | MUC6 | PIK3CA | ROS1 | TSC2 |  |

**Table S2.** The 520 cancer-related genes included in the OncoScreen Plus panel

| ABL1 | BCL2L1 | CDKN2B | EGFL7 | FAS | HIST1H3C | JAK1 | MEF2B | NRG1 | PMS1 | RBM10 | SOX2 | TRAF2 |
| --- | --- | --- | --- | --- | --- | --- | --- | --- | --- | --- | --- | --- |
| ABL2 | BCL2L11 | CDKN2C | EGFR | FAT1 | HIST1H3D | JAK2 | MEN1 | NSD1 | PMS2 | RECQL4 | SOX9 | TRAF7 |
| ACVR1 | BCL2L2 | CEBPA | EIF1AX | FAT3 | HIST1H3E | JAK3 | MET | NTHL1 | PNRC1 | REL | SOX10 | TRRAP |
| ACVR1B | BCL6 | CENPA | EIF4A2 | FBXW7 | HIST1H3F | JUN | MGA | NTRK1 | POLD1 | RET | SOX17 | TSC1 |
| ADGRA2 | BCOR | CHD1 | EIF4E | FCGR2B | HIST1H3G | KAT5A | MITF | NTRK2 | POLE | RFWD2 | SPEN | TSC2 |
| AKT1 | BCORL1 | CHD2 | ELOC | FGF10 | HIST1H3H | KDM5A | MLH1 | NTRK3 | POM121L12 | RHEB | SPOP | TSHR |
| AKT2 | BCR | CHD4 | EMSY | FGF12 | HIST1H3I | KDM5C | MLH3 | NUP93 | PPM1D | RHOA | SPTA1 | U2AF1 |
| AKT3 | BLM | CHEK1 | EP300 | FGF23 | HIST1H3J | KDM6A | MPL | PAK1 | PPP2R1A | RICTOR | SRC | VEGFA |
| ALK | BMPR1A | CHEK2 | EPCAM | FGF6 | HIST2H3C | KDR | MRE11A | PAK3 | PPP2R2A | RIT1 | SRSF2 | VEGFB |
| ALOX12B | BRAF | CHUK | EPHA2 | FGF7 | HIST2H3D | KEAP1 | MSH2 | PAK7 | PPP6C | RNF43 | STAG2 | VEGFC |
| AMER1 | BRCA1 | CIC | EPHA3 | FGFR14 | HIST3H3 | KEL | MSH3 | PALB2 | PRDM1 | ROS1 | STAT3 | VHL |
| ANKRD11 | BRCA2 | CRBN | EPHA5 | FGFR1 | HLA-A | KIT | MSH6 | PARK2 | PREX2 | RPA1 | STAT4 | VTCN1 |
| APC | BRD4 | CREBBP | EPHA7 | FGFR2 | HNF1A | KLF4 | MST1 | PARP1 | PRKAR1A | RPS6KA4 | STAT5A | WISP3 |
| APCDD1 | BRIP1 | CRKL | EPHB1 | FGFR3 | HNF1B | KLHL6 | MST1R | PARP2 | PRKC1 | RPS6KB2 | STAT5B | WRN |
| AR | BTG1 | CRLF2 | ERBB2 | FOXA1 | HOXB13 | KMT2A | MTOR | PARP3 | PRKDC | RPTOR | STK11 | WT1 |
| ARAF | BTK | CSF1R | ERBB3 | FOXL2 | HRAS | KMT2C | MUTYH | PARP4 | PRSS8 | RUNX1 | STK40 | XIAP |
| ARFRP1 | CALR | CSF3R | ERBB4 | FRS2 | HSD3B1 | KMT2D | MYC | PAX5 | PTCH1 | RUNX1T1 | SUFU | XPO1 |
| ARID1A | CARD11 | CTCF | ERBB5 | FYN | HSP90AA1 | KRAS | MYCL | PBRM1 | PTEN | RYBP | SUZ12 | XRCC2 |
| ARID1B | CASP8 | CTLA4 | ERCC1 | GABRA6 | ICOSLG | LATS1 | MYCN | PDCD1 | PTK2 | SDHA | SYK | XRCC3 |
| ARID2 | CBFB | CTNNA1 | ERCC2 | GATA4 | ID3 | LATS2 | MYD88 | PDCD1LG2 | PTPN11 | SDHAF2 | TACC3 | YAP1 |
| ARID5B | CBL | CTNNB1 | ERCC3 | GATA6 | IDH1 | LMO1 | MYOD1 | PDFRA | PTPRD | SDHB | TAF1 | YES1 |
| ASXL1 | CCND1 | CUL3 | ERCC4 | GID4 | IDH2 | LRP1B | NBN | PDGFRB | PTPRS | SDHC | TBX3 | ZBTB2 |
| ASXL2 | CCND2 | CUL4A | ERCC5 | GNA13 | IFNGR1 | LYN | NCOA3 | PDK1 | PTPRT | SDHD | TCF3 | ZFHX3 |
| ATF1 | CCND3 | CUL4B | ERG | GPS2 | IGF1 | LZTR1 | NCOR1 | PGR | QK1 | SETD2 | TCF7L2 | ZNF217 |
| ATM | CCNE1 | CXCR4 | ERRFI1 | GREM1 | IGF1R | MAG12 | NEB | PHOX2B | RAB35 | SF3B1 | TERC | ZNF703 |
| ATR | CD274 | CYCLD | ESR2 | GRM3 | IGF2 | MALT1 | NEGR1 | PIK3CA | RAC1 | SH2B3 | TERT | ZNRF3 |
| ATRX | CD276 | CYP17A1 | EWSR1 | GSK3B | IKBKE | MAP2K1 | NF1 | PIK3CB | RAD21 | SH2D1A | TET1 | ZRSR2 |
| AURKA | CD79A | DAXX | EZH2 | GSTM1 | IKZF1 | MAP2K2 | NF2 | PIK3C2B | RAD50 | SHQ1 | TET2 |  |
| AURKB | CD79B | DCUN1D1 | FAM175A | GSTT1 | IL10 | MAP2K4 | NFE2L2 | PIK3C2G | RAD51 | SLIT2 | TGFBR1 |  |
| AXIN1 | CDC73 | DDR2 | FAM46C | H3F3A | IL7R | MAP3K1 | NFKB1A | PIK3C3 | RAD51B | SLX4 | TGFBR2 |  |
| AXIN2 | CDH1 | DICER1 | FANCA | H3F3B | INHA | MAP3K13 | NKX2-1 | PIK3CD | RAD51C | SMAD2 | TIPARP |  |
| AXL | CDK12 | DIS3 | FANCC | HDAC1 | INHBA | MAP3K14 | NKX3-1 | PIK3CG | RAD51D | SMAD3 | TMEM127 |  |
| B2M | CDK4 | DNAJB1 | FANCD2 | HDAC2 | INPP4A | MAP3K3 | NOTCH1 | PIK3R1 | RAD52 | SMAD4 | TMPR552 |  |
| BACH1 | CDK6 | DNMT1 | FANCE | HDAC4 | INPP4B | MAPK1 | NOTCH2 | PIK3R2 | RAD54L | SMARCA4 | TNFAIP3 |  |
| BAP1 | CDK8 | DNMT3A | FANCF | HGF | INSR | MAX | NOTCH3 | PIK3R3 | RAF1 | SMARCB1 | TNFRSF14 |  |
| BARD1 | CDKN1A | DNMT3B | FANCG | HIST1H1C | IRF2 | MCL1 | NOTCH4 | PIM1 | RANBP2 | SMARCD1 | TNFSF11 |  |
| BBC3 | CDKN1B | DOT1L | FANCI | HIST1H2BD | IRF4 | MDM2 | NPM1 | PLCG2 | RARA | SMO | TOP1 |  |
| BCL2 | CDKN1C | E2F3 | FANCL | HIST1H3A | IRS1 | MDM4 | NRAS | PLK2 | RASA1 | SNCAIP | TOP2A |  |
| BCL10 | CDKN2A | EED | FANCM | HIST1H3B | IRS2 | MED12 | NR4A3 | PMAIP1 | RB1 | SOCS1 | TP53 |  |

**Table S3.** Mutation types detected among the patients in the cohort based on molecular subtype

| Mutation type | All | TNBC | HR+/HER2+ | HR+/HER2- | HR-/HER2+ | Unknown |
| --- | --- | --- | --- | --- | --- | --- |
| Missense | 475 | 202 | 37 | 142 | 63 | 31 |
| Nonsense | 76 | 26 | 7 | 31 | 10 | 2 |
| Splice site | 61 | 35 | 3 | 16 | 7 | 0 |
| Nonsynonymous variants detected in introns | 42 | 22 | 6 | 9 | 3 | 2 |
| Small insertion deletion (including disruptive indels) | 22 | 11 | 0 | 7 | 3 | 1 |
| Frameshift | 101 | 49 | 5 | 34 | 9 | 4 |
| Fusion | 23 | 8 | 1 | 7 | 7 | 0 |
| Copy number deletion | 60 | 30 | 0 | 17 | 1 | 12 |
| Copy number amplification | 341 | 115 | 33 | 135 | 41 | 17 |
| Total | 1,201 | 498 | 92 | 398 | 144 | 69 |

**Table S4.** Distribution of pathogenic/likely pathogenic germline and somatic BRCA1/2 mutations detected from the cohort

|  | Germline BRCA1/2 | Somatic BRCA1/2 | Somatic + Germline BRCA1/2 |
| --- | --- | --- | --- |
| HR-/HER2+ (n=36) | 0 (0.0%) | 0 (0.0%) | 0 (0.0%) |
| HR+/HER2+ (n=20) | 0 (0.0%) | 0 (0%) | 0 (0.0%) |
| HR+/HER2- (n=91) | 5 (5.5%) | 1 (1.1%) | 6 (6.6%) |
| TNBC (n=137) | 6 (4.4%) | 5 (3.6%) | 11 (8.03%) |
| Unknown (n=6) | 0 (0.0%) | 0 (0.0%) | 0 (0.0%) |
| Total (n=290) | 11 (3.8%) | 6 (2.1%) | 17(5.9%) |
