# Supplementary figures and images for: Characterizations of Cancer Gene Mutations in Chinese Metastatic Breast Cancer Patients
Source: Front Oncol. 2020 Jun 30;10:1023. doi: 10.3389/fonc.2020.01023 (PMC7338574; doi:10.3389/fonc.2020.01023)

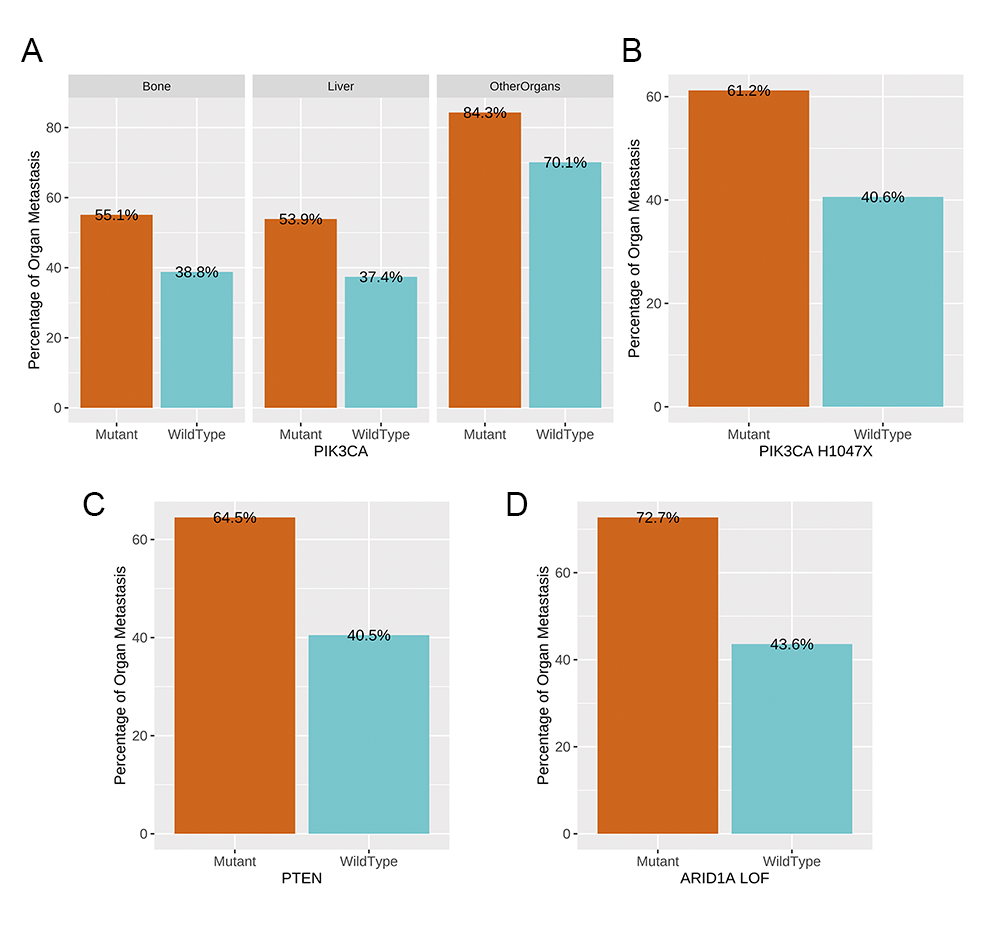

Supplement: Supplementary file 2 [file Image_1.TIF]
